# Evaluation of Oxidative Stress before and after Using Laser and Photoactivation Therapy as Adjuvant of Non-Surgical Periodontal Treatment in Patients with Rheumatoid Arthritis

**DOI:** 10.3390/antiox10020226

**Published:** 2021-02-03

**Authors:** Maria-Alexandra Martu, Petra Surlin, Luminita Lazar, George Alexandru Maftei, Ionut Luchian, Dorin-Nicolae Gheorghe, Elena Rezus, Vasilica Toma, Liliana-Georgeta Foia

**Affiliations:** 1 “Grigore T. Popa”, University of Medicine and Pharmacy, 16 Universitatii Str., 700115 Iasi, Romania; maria-alexandra.martu@umfiasi.ro (M.-A.M.); ionut.luchian@umfiasi.ro (I.L.); elena.rezus@umfiasi.ro (E.R.); vasilica.toma@umfiasi.ro (V.T.); georgeta.foia@umfiasi.ro (L.-G.F.); 2 Department of Periodontology, University of Medicine and Pharmacy of Craiova, 2 Petru Rares Str., 200349 Craiova, Romania; dorin.gheorghe@umfcv.ro; 3 “George E. Palade”, University of Medicine, Pharmacy, Science and Technology, 38 Gh. Marinescu Str., 540139 Targu-Mures, Romania; luminita.lazar@umftgm.ro

**Keywords:** oxidative stress, periodontitis, rheumatoid arthritis, laser, photodynamic therapy

## Abstract

(1) Background: The aim of this split-mouth design study was to analyze the clinical periodontal indexes and oxidative stress markers in gingival crevicular fluid modifications after three periodontal disease treatment possibilities (scaling and root planning—SRP; SRP and diode laser—L; SRP and photodynamic therapy—PDT). (2) Methods: The study was conducted on 52 patients: systemically healthy subjects with periodontal disease—non-RA (*n* = 26); and test group (*n* = 26) subjects with rheumatoid arthritis and periodontal disease—RA. Clinical periodontal measurements (probing depth—PD; Löe and Silness gingival index—GI; papillary bleeding index—PBI; and periodontal community index of treatment needs—CPITN) and oxidative stress markers (8-hydroxy-2’-deoxyguanosine (8-OHdG) and 4 hydroxynonenal (4-HNE)) were analyzed at baseline (T0), after three sessions of periodontal treatment (T1), and 6 months after treatment (T2). (3) Results: Periodontal therapy improved clinical periodontal measurements and oxidative stress markers in both analyzed groups, with supplementary benefits for laser- and PDT-treated periodontal pockets. (4) Conclusions: The analyzed oxidative stress markers decreased significantly following non-surgical periodontal therapy in both rheumatoid arthritis and systemically healthy patients. All the periodontal disease treatment possibilities analyzed in this study offered clinical and paraclinical improvements; however, the association of laser with SRP and photodisinfection with SRP yielded the best clinical and paraclinical outcomes when compared to SRP alone.

## 1. Introduction

Oxidative stress is an alteration of the balance between the levels of oxidizing agents and those of antioxidants. During normal cellular metabolic processes, free radicals and reactive metabolites are continuously generated. When the rate of oxidant production exceeds the capacity of antioxidant systems to eliminate oxidizing products, oxidative stress is installed, which subsequently leads to cell and tissue damage [1].

The dual link between periodontal disease and rheumatoid arthritis (RA) has recently been described in the scientific literature and oxidative stress seems to be an important component of this relationship [2]. Mitochondria are central regulators of cellular metabolism and major sources of intracellular of reactive oxygen species (ROS), and increase in their production has been described in both subjects with periodontal disease and those with rheumatoid arthritis [1]. Recently, ROS production has been identified as a first step in activating the inflammatory response, stimulating the production of pro-inflammatory cytokines through mitochondria, which may have multiple critical roles in immunity [1].

Tissue destruction in periodontitis is considered to be the result of an excessive inflammatory response to bacterial plaque; this leads to the release of reactive oxygen species, such as hydrogen peroxide and superoxide anion from neutrophils, with subsequent setup of the oxidative stress event [3].

Periodontal disease therapy has a complex course and current treatment models involve stratifying patients to allow practitioners to individualize therapy according to the complexity of the pathology but also in terms of systemic integration of risk assessment for each patient, such as a systemic disease which modifies the response to inflammation (rheumatoid arthritis, diabetes, metabolic syndrome, chronic kidney disease). Certain subjects have a more severe and rapidly progressive form and therefore, require intensive monitoring and adjunctive treatment (doxycycline in a sub-antimicrobial dose, laser, photodisinfection) or even surgical therapy as opposed to a patient with a moderate form of periodontitis [4].

Several oxidative stress biomarkers have been explored in the literature as potential useful parameters in monitoring the progression of both periodontitis and rheumatoid arthritis. Biomarkers of oxidative stress (derived from protein damage, lipids, uric acid, and DNA oxidation) were consistently and significantly higher in value in patients with rheumatoid arthritis compared to systemically healthy individuals, this increase being observed in serum, plasma, urine, synovial fluid, and whole blood [5].

In order to determine the oxidative stress as comprehensively as possible, one must look at as many compartments that cause oxidative stress as possible. For example, 8-Hydroxy-deoxyguanosine (8-OHdG) results from the metabolization of nucleic acids, thus being a marker of DNA damage; on the other hand, 4 hydroxynonenal (4 HNE) is a marker of lipid peroxidation [1].

The aim of this split-mouth design study was to analyze clinical periodontal modifications (probing depth—PD; Löe and Silness gingival index—GI; papillary bleeding index—PBI; and periodontal community index of treatment needs—CPITN) and oxidative stress (8-hydroxy-2’-deoxyguanosine—8-OHdG; and 4 hydroxynonenal—4 HNE) markers after three periodontal disease treatment techniques (scaling and root planning—SRP; SRP and laser; SRP and photodisinfection) in patients with rheumatoid arthritis and periodontal disease and in systemically healthy subjects affected by periodontal disease.

## 2. Materials and Methods

### 2.1. Study Group

This study was carried out between April 2017 and November 2018. According to the Recovery Hospital, Iași Registry, out of 1108 registered cases of rheumatoid arthritis in 2017, 182 were under treatment with biologic disease-modifying antirheumatic drugs. In a previous study, it was reported that rheumatoid arthritis patients were 37% more likely to have periodontitis [6]. Thus, the minimum sample size in a population of 67 patients with a margin of error of 15 and 95% probability was 26 patients. We also included 26 patients with periodontal disease but without rheumatoid arthritis.

When performing power analysis on the 2 groups, with 80% power for 1mm difference in probing depth between the two groups, type I error of 0.05, and type II of 0.2, a total of 16 patients per group were needed.

A total of 143 subjects agreed to participate in the study; however, following the inclusion and exclusion criteria, only 63 subjects were selected, of which only 52 patients completed the study.

Following the obtaining of ethics approval for this study from the research ethics commission within the University of Medicine and Pharmacy “Grigore T. Popa” Iași, informed consent was obtained from all patients included in the study.

The subjects have been divided into two groups as follows: the systemically healthy group (non-RA) consisting of 26 patients with periodontal disease but systemically healthy, recruited from the Clinical Base of Dental Education “M. Kogălniceanu” from the “Grigore T. Popa” University of Medicine and Pharmacy, Iași, and the rheumatoid arthritis group (RA group), consisting of 26 patients with rheumatoid arthritis and periodontal disease with medical evidence at the Recovery Hospital in Iași.

The inclusion criteria in the study were based on the clinical diagnosis of periodontitis, probing depth ≥ 4 mm for at least 2 sites/hemiarch, at least one tooth of probing depth ≥ 4 mm in a minimum of 3 hemiarches, and the presence of at least 10 teeth in the oral cavity. For the rheumatoid arthritis group, the inclusion criteria consisted of a defined medical diagnosis of rheumatoid arthritis (American College of Rheumatology) and treatment with biologic disease-modifying antirheumatic drugs [7].

The exclusion criteria consisted of: use of anti-inflammatory therapy in the last 3 months (except for the group with rheumatoid arthritis), use of antibiotics in the last 12 months, periodontal therapy in the last 12 months, and the presence of mental disorders, systemic diseases (except for the group with RA), smoking, pregnancy, or lactation.

### 2.2. Periodontal Examination

In each subject, we performed a full medical anamnesis, evaluating systemic status and current medication.

The periodontal clinical examination was performed by a single calibrated periodontal specialist who assessed the following periodontal health indices: probing depth (PD), Löe and Silness gingival index (GI), papillary bleeding index (PBI), and periodontal community periodontal index of treatment needs (CPITN). Periodontal probing was performed using a UNC 12-type probe in 6 sites per tooth, buccal mesial–central–distal and oral mesial–central–distal. The clinical diagnosis of periodontal disease was assigned according to the 2018 classification of periodontal diseases and conditions [8].

### 2.3. Gingival Crevicular Fluid Sampling

After initial clinical examination of the oral cavity, we established the dental units chosen for the study—the deepest periodontal pockets in each hemiarch.

Crevicular fluid (GCF) samples were harvested through the absorption technique, the intracrevicular method, using PerioPaper Strips^®^ (Oraflow, New York, NY, USA), from 3 sites in each patient and collected three times: T0—before periodontal treatment; T1—after 3 therapy sessions; T2—6 months after the end of periodontal therapy.

Before sampling, patients were asked to rinse their mouth vigorously twice with water.

Each tooth was isolated with sterile cotton rolls and air dried; with another sterile cotton ball, the supragingival plaque was removed, after which the periopaper was inserted into the gingival sulcus, at the deepest point for 30 s. Strips contaminated with saliva or blood were not used. After collection, GCF volume was determined using a calibrated Periotron 8000, and then the sample was placed in a 1.5 mL cryotube containing 1 mL of phosphate buffer (PBS) and stored at −80 °C until the analysis of biological markers.

### 2.4. Periodontal Treatment

On the first day, clinical parameters were recorded for each patient, and crevicular fluid samples were collected from 3 of the deepest areas, each from a different quadrant. Scaling and root planning (SRP) was performed with an ultrasonic device (Hu-Friedy—Symmetry Iq^®^ 4000 Piezo Scaler) and Gracey curettes. The patient was recalled the next day and 2 sites out of the 3 previously designated were randomly selected for laser treatment or photodisinfection, which was applied once a week for a total of 3 treatment sessions.

The sites assigned to a therapy group were marked with C (control site that will receive conventional periodontal therapy only), PDT (conventional therapy + antimicrobial photodynamic disinfection), and L (conventional therapy + laser).

For the laser group, we used a 940 nm Biolase^®^ Epic X diode, with a tip of 300 mm in diameter, uninitialized continuous mode of operation, 1 W for 30 s.

For the PDT group, we utilized antimicrobial photodynamic therapy through a Helbo^®^ TheraLite (Bredent medical) Photodynamic Systems GmbH & Co KG device, wavelength 670 nm, output 75 mW/cm^2^, spot size of 0.06 cm in diameter, with the photosensitizer provided by the manufacturer (3,7-bis phenothiazine-5-ium chloride). Patients were instructed to rinse their mouth vigorously twice with water, followed by subsequent application of the photosensitizer at the designated site, left to act for 60 s. The patient was instructed to rinse their mouth once more to remove excess substance, and the light source was applied for 60 s.

After a period of 4 weeks from the first visit, the patient was called for follow-up and was examined clinically and immunobiochemically for modifications; then, at 6 months, the final verification was performed.

### 2.5. Oxidative Stress Marker Assessment

All samples were analyzed consecutively after being gently defrosted and homogenized for 30s and centrifuged for 5 min at 1500 rpm to elude. By means of immunoenzymatic protocols (ELISA), we analyzed: 8-hydroxy-2’-deoxyguanosine (8-OHdG) and 4 hydroxynonenal (4-HNE). The reagents used were: the Human 8-OHdG ELISA kit (My BioSource) and the Human 4-HNE (4-Hydroxinonenal) ELISA kit (My BioSource).

All reagents, samples, and standards were prepared according to the instructions provided by the manufacturer for the ELISA method.

### 2.6. Statistical Analysis

All statistical analyses were performed using SPSS version 24.0 software (IBM Corp., Armonk, NY, USA). The tests of normality in frequentist statistics, skewness and kurtosis tests (−2 < *p* < 2), were used to examine the distribution of continuous variables. For multiple comparisons of normal distributed series of values, a post hoc Bonferroni test was applied after one way ANOVA. Associations between categorical variables were assessed by Chi-square tests. Associations between continuous variables were analyzed using Pearson two-tailed correlation coefficients.

## 3. Results

The demographic characteristics at baseline of each study group are briefly stated in Table 1.

Compared with non-RA patients, the RA group is characterized by a significantly higher mean level of PD (5.40 vs. 5.02; *p* = 0.001) and a significantly lower frequency of patients with PBI index 3 and 4 (68% vs. 92.3%; 0.001), and therefore, a lower bleeding index compared to the systemically healthy group (Table 1). GI, CPITN, and number of teeth left did not show statistically significant differences for our two study groups.

### 3.1. Clinical Periodontal Indexes

At baseline, probing depth (PD) was significantly lower in the non-RA group compared to the RA group, whereas PBI was significantly higher (*p* < 0.05) (Table 1). Periodontal probing depth decreased significantly after periodontal treatment in all analyzed periods (*p* = 0.001), for both study groups. At the end of the study, in T2, all clinical periodontal measurements were upgraded, while considering periodontal treatment strength, the outclass improvements were observed for laser and photodisinfection adjunctive treated sites (Table 2).

In the RA group, control sites at T0 have a significantly higher mean PD level than non-RA patients (5.87 vs. 5.38; *p* = 0.007), which is maintained at T1 (5.29 vs. 4.82 *p* = 0.013) and T2 (5.03 vs. 4.51 *p* = 0.005).

In evolution, depending on the treatment, the percentage of distribution of teeth according to periodontal clinical indexes highlights the migration of higher to lower values of periodontal indexes.

In control sites (SRP only) at baseline, 61.5% of sites registered a GI index = 3 and 30.8% a GI index of 4. At T1 and T2, respectively, GI decreased, registering values of 2 (46.2 % and 42.3%, respectively) and 3 (46.2% and 38.5%, respectively) (*p* = 0.001). Regarding PBI index, at T0, 69.2% of control sites had values of 3 and 4, while at T1, the values for this index decreased to 1 and 2 in 69.2% of sites (*p* = 0.001), this percentage for 1 and 2 values increasing even more at 73% in T2 (*p* = 0.001). At baseline, 53.8% of sites had a CPITN of 4, this value decreasing to 7.7% at T1, and at T2, no teeth were included in this value (*p* = 0.001).

In laser-treated sites at T0, 38.5% had a GI value of 3 and 57.7% of 4, respectively. At T1 and T2, the values shifted to GI = 1 (23.1 % and 46.2%, respectively) and GI = 2 (53.8% and 46.2%, respectively) (*p* = 0.001). When analyzing the PBI index, at T0, 60.5% of sites registered values of 3–4, while at T1, 68.5% of the teeth were at 1–2 (*p* = 0.001); finally, at T2, 84.6% of sites had values of 1–2 (*p* = 0.001). At T0, 53.8% of sites had a CPITN index of 4; at T1, these value decreased to 19.2%, and at T2, no sites were identified in this classification (*p* = 0.001).

In PDT-treated sites at T0, 38.5% of periodontal pockets had GI values of 3 and 53.8% values of 4, respectively; at T1 and T2, GI values decreased to 1 (19.2 % and 46.2%, respectively) and 2 (46.2% and 53.8%, respectively) (*p* = 0.001). At baseline, PBI index values of 3–4 had a proportion of 73.1%, while at T1 and at T2, respectively, values of 1 and 2 were significantly more prevalent (92.3% and 88.4%, *p* = 0.001). At T0, 61.5% of sites had CPITN values of 4; in evolution, these values registered a decrease, only 11.5% for code 4 in T1, and none in T2 (*p* = 0.001).

### 3.2. 8-OHdG (8-Hydroxy-2’-Deoxyguanosine)

The evaluation of the mean level of 8-OHdG at the beginning of the study (T0) pointed out mean levels significantly higher in the RA group subjects than those recorded in the non-RA group (*p* = 0.001).

At T1, for both groups, the mean levels decreased significantly subsequent to therapy, the lowest mean level of 8-OHdG being observed in the laser-treated sites, with the highest mean level in the control sites (15.18 vs. 5.97; 6.19; *p* = 0.001 and 14.96 vs. 6.18; 6.30; *p* = 0.001, respectively). After 6 months, in T2, the lowest mean values of 8-OHdG in the RA group were noted in the laser and photodisinfection sites, and the highest mean level in the SRP only sites (3.46; 3.46 vs. 8.04; *p* = 0.001); in the non-RA group, the lowest mean level was detected in the photoactivated periodontal sites and the highest mean level in the control (3.49; 3.42 vs. 8.29; *p* = 0.001) (Table 3).

### 3.3. 4-Hydroxynonenal (4-HNE)

Evaluation of the mean level of 4-HNE at the beginning of the study (T0) did not reveal any significant differences between the analyzed groups (*p* = 0.177). Subsequently, after three therapy sessions, at T1, the mean levels decreased significantly in both groups (*p* = 0.001). In rheumatoid arthritis patients, the lowest mean level of 4-HNE was observed in sites treated with laser, and the highest mean level was observed in group C (14.35 vs. 6.48; 7.29; *p* = 0.001), as opposed to the systemically healthy subjects, which had the best results with the photodisinfection therapy (28.53 vs. 24.89; 24.41; *p* = 0.001); intergroup, the mean levels of 4-HNE at T1 were significantly lower in the RA group compared to the non-RA group: C (14.35 vs. 28.53; *p* = 0.001); L (6.48 vs. 24.89; *p* = 0.001); PDT (7.29 vs. 24.41; *p* = 0.001).

Evaluation of the mean level of 4-HNE at T2 revealed significantly lower values in the RA group than those recorded in the non-RA group: C (3.18 vs. 18.49; *p* = 0.001); L (1.41 vs. 13.05; *p* = 0.001); PDT (1.48 vs. 12.25; *p* = 0.001). For the RA group, the lowest mean level of 4-HNE was observed in the PDT-treated periodontal pockets, the value being relatively similar to that recorded in group L, and the highest mean level in group C (1.81; 1.48 vs. 3, 18; *p* = 0.001); in the non-RA group, the lowest mean level of 4-HNE was also observed in the PDT group, and the highest in group C (12.25; 13.05vs 18.49; *p* = 0.001) (Table 4).

At the end of the study in the RA group, the mean levels decreased significantly, in all treated sites, the most powerful decrease being recorded both for 8-OHdG and 4-HNE in PDT sites (91, 6% vs. 91.7%; *p* = 0.001 and 94.5% vs. 95.6%; *p* = 0.001, respectively). For systemically healthy patients, in sites treated with laser or photoactivation, there were significant decreases for both markers 8-OHdG and 4-HNE, especially in the group with the photoactivation technique (88.9% vs. 89.2%; *p* = 0.001) and (59.6% vs. 56%; *p* = 0.001), respectively.

### 3.4. Association between Clinical Periodontal Indexes and Oxidative Markers

In the analyzed rheumatoid arthritis patients, the individual values of 8-OHdG and 4-HNE did not significantly correlate with probing depth in any of the analyzed times.

In both groups, the individual values of 8-OHdG did not correlate significantly with PD: RA group (T0: r = +0.101; *p* = 0.381; T1: r = +0.056; *p* = 0.625; T2: r = +0.075; *p* = 0.351) and non-RA group (T0: r = +0.309; *p* = 0.056; T1: r = −0.140; *p* = 0.220; T2: r = +0.050; *p* = 0.661), respectively. Furthermore, the individual values of 4-HNE also did not significantly correlate with PD in either of the studied groups: RA group (T0: r = +0.222; *p* = 0.151; T1: r = −0.006; *p* = 0.955; T2: r = −0.280; *p* = 0.156) and non-RA group (T0: r = +0.229; *p* = 0.144; T1: r = −0.032; *p* = 0.783; T2: r = −0.100; *p* = 0.384) (Figure S1 and S2).

Gingival index correlated statistically significantly with 8-OHdG and 4-HNE only 6 months after treatment in the RA and non-RA groups (*p* = 0.001, *p* = 0.05). Papillary bleeding index correlated significantly in T1 and T2 with 8-OhdG (*p* = 0.017 and *p* = 0.001, respectively) and only in T2 with 4-HNE (*p* = 0.003) in the RA group, whereas in the non-RA group, only 8-OHdG correlated with PBI in all analyzed periods (*p* = 0.035, *p* = 0.007, *p* = 0.003). In the RA group, CPITN significantly correlated with 8-OHdG only 6 months after treatment (*p* = 0.005) and with 4-HNE in T1 and T2 (*p* = 0.017 and *p* = 0.001), whereas for the non-RA subjects, CPITN correlated with 8-OHdG (*p* = 0.023) and 4-HNE (0.043) at baseline and only with 8-OhdG at 6 months after periodontal treatment (*p* = 0.001) (Table 5).

## 4. Discussion

It is commonly accepted that oxidative stress has a contribution to the onset and progression of most oral conditions and especially periodontal disease [9]. This process plays a key role in the progression of chronic inflammation, degradation of the extracellular matrix of the periodontium, and bone remodeling. In addition, oxidative stress is an important source of oral inflammation [10]. It has been shown that the rate of ROS production in the oral cavity is determined by the number and functional status of neutrophils participating in phagocytosis. In addition, overproduction of ROS by neutrophils can cause collagen breakdown, disorders in proteoglycan synthesis, and depolymerization of hyaluronic acid, thus triggering loss of periodontal tissue integrity and of its physiologic properties [11].

Moreover, oxidative stress is one of the main factors responsible for damage to the salivary glands during metabolic diseases (obesity, type 1 and 2 diabetes), autoimmune disorders (Sjögren’s disease, rheumatoid arthritis, systemic scleroderma), neurodegenerative diseases (dementia, Alzheimer’s disease), oral cancer, and oral precancerous conditions (keratosis) [12].

It is important to note that neutrophils present in the oral cavity, unlike peripheral neutrophils, generate superoxide anions and nitrogen oxide in the absence of any stimuli. ROS formed in this process are responsible for the elimination of pathogenic microorganisms but can also impair the surrounding tissues by oxidative modification of the host cell elements [13].

Increased oxidative stress has been associated with enhanced lipid peroxidation in patients with RA. Lipid peroxidation occurs as a result of increased oxidative stress induced by alteration of the pro/antioxidant balance and is an important pathological process in oxygen toxicity. Levels of conjugated dienes, isoprostanes, 4-HNE, and malondialdehyde have been shown to increase subsequent to lipid peroxidation. On the other hand, the etiology of structural lesions such as bone erosion and joint deformity has not been fully understood in RA [5]. Cartilage and bone damage is associated with the activation of free radicals, pro-inflammatory cytokines, and metalloproteinases. Inflammatory cells such as macrophages, T cells, B cells, and neutrophils accumulate in the inflamed synovial membrane. For these reasons, in recent years, researchers have focused on oxidative stress and the increased tendency of patients with RA to lipid peroxidation [14].

Although various studies on antioxidant enzymes and vitamins have been performed in inflammatory diseases, a relatively limited number of studies have focused on lipid peroxidation status and inflammatory parameters in patients with RA. The determination of the pathological mechanism is of particular importance in the early diagnosis and treatment of RA, in order to improve the quality of life of these patients.

Patients with chronic periodontitis possess a diminished antioxidant capacity compared to periodontally healthy patients [15]. Although total antioxidant capacity is not a specific marker of antioxidant potential, uric acid, glutathione peroxidase, and reduced glutathione as specific antioxidants have been reported to be significantly lower in the saliva of patients with chronic or aggressive periodontitis. Lactoferrin, myeloperoxidase, and IL-1β have all been positively correlated with clinical markers of periodontal involvement [16].

Periodontitis has been confirmed to be associated with the hyperactivity of peripheral blood neutrophils, which should be the predominant source of ROS. Reports suggest that neutrophil hyperactivity is likely to be a host immune response to periodontitis inflammation, which could be genetically predisposed. Numerous studies have suggested that periodontitis may contribute to both local and systemic oxidative stress [11].

ROS can react with DNA and cause damage to purine and pyrimidine nitrogenous bases. 8-Hydroxy-deoxyguanosine (8-OHdG), a product of nucleotide metabolism from nucleic acids, is the biomarker most often used to determine DNA destruction induced by oxidative stress, although it does not accurately reflect all DNA impairment triggered by oxidative stress [17].

Plentiful research studies have indicated a higher level of 8-OHdG in GCF and saliva of patients with periodontitis compared to healthy ones, as well as a significant association with periodontal clinical parameters [18,19,20,21]; in addition, levels are significantly reduced by periodontal treatment [19,22] but with no difference between local levels of 8-OHdG in people with gingivitis and periodontitis, as well as among patients with aggressive and chronic periodontitis [20]. Several studies have indicated that the level of 8-OHdG is directly related to the presence and/or amount of bacteria such as *Porphyromonas gingivalis*, *Tannerella forsythia*, *Treponema denticola*, and *Streptococcus anginosus* [23,24]. Moreover, studies investigating serum 8-OHdG concentrations have demonstrated their possible shift by several systemic conditions such as obesity and hyperlipidemia [25,26].

In our studied groups, patients with rheumatoid arthritis had a significantly higher mean level of PD (5.40 vs. 5.02; *p* = 0.001), but lower papillary bleeding index values compared to the non-RA group; on the other hand, GI, CPITN, and number of teeth left did not show statistically significant differences for the two study groups. Mean baseline 8-OHdG levels were significantly higher in the RA group than those in the non-RA group. Following periodontal therapy, the level of this marker decreased significantly, with best outcomes following photoactivation therapy, in both groups.

A meta-analysis revealed redoubling of the 8-OHdG level in the saliva of subjects with chronic periodontitis compared to periodontally healthy controls, shifting toward this product of purine base degradation as a really faithful marker for the assessment of periodontal alteration implications [17].

Lipid peroxidation triggered by free radicals results in changes in the structural and functional integrity of cell membranes. Several lipid peroxidation products such as malondialdehyde (MDA), 4-HNE, and isoprostane have been used to assess both local and systemic oxidative damage associated with periodontitis [27,28]. 4-HNE is a major aldehyde end product associated with lipid peroxidation, but data on this biomarker in periodontitis are limited for now. A study by Hendek et al. investigated the impact of periodontitis, smoking, and periodontal treatment on 4-HNE levels in GCF, saliva, and serum, and reported significantly different levels in GCF between periodontitis smokers and periodontally healthy non-smokers [18]. In contrast to this study, Onder et al. in 2017 showed that 4-HNE levels are elevated in periodontal disease only in serum, but not in saliva. None of the above studies appreciated the change in the level of 4-HNE post periodontal therapy [26].

At baseline, 4-HNE did not record statistically significant differences between the two groups; however, the values of this marker were significantly reduced post therapy for both rheumatoid arthritis and systemically healthy subjects. The lowest mean level of 4-HNE was observed at the sites treated by photodynamic therapy, with values quite close to those recorded at the sites where the protocol included a diode laser, while the highest mean level was noticed at sites treated only with scaling and root planning.

In a recent study, Balogh et al. indicated increased secretion of proangiogenic and proinflammatory mediators released by synovial fibroblasts harvested from synovial fluid in patients with primary rheumatoid arthritis following stimulation with 4-HNE. Moreover, proangiogenic processes have been potentiated, including invasion, proliferation and migration of endothelial cells, formation of tubular structures, and secretion of proangiogenic mediators. These findings provide evidence for direct and indirect proangiogenic effects in response to 4-HNE in the inflamed joint. The authors of this study conclude that under inflammatory conditions, oxidative stress can mediate angiogenic mechanisms through pathways independent of vascular endothelial growth factor (VEGF), involving ROS-induced lipid oxidation [29].

The activity of antioxidant enzymes is altered through SRP, thus supporting the hypothesis which emphasizes the role of oxidative stress in periodontal disease initiation and progression. Moreover, a negative correlation was observed between the periodontal parameters studied and salivary antioxidant levels, probably because as the periodontal condition deteriorates due to ROS production, antioxidants are spoiled due to being used to maintain a balance and thus, they decline [30].

When analyzing the correlation between salivary values of oxidative markers and periodontal indexes, in the rheumatoid arthritis patients included in our study, we could observe that the individual values of 8-OHdG and 4-HNE did not significantly correlate with probing depth in either of the analyzed times. Gingival index correlated statistically significantly with 8-OHdG and 4-HNE only 6 months after treatment in the analyzed groups (*p* = 0.001, *p* = 0.05). Papillary bleeding index correlated significantly with 8-OHdG after three sessions of periodontal treatment and 6 months after (*p* = 0.017 and *p* = 0.001), whereas with 4-HNE only 6 months after periodontal therapy (*p* = 0.003); on the other hand, in the systemically healthy group, only 8-OHdG correlated with PBI in all analyzed instances (*p* = 0.035, *p* = 0.007, *p* = 0.003), while 4-HNE did not correlate in any of the sampling moments with PBI. In the RA group, CPITN significantly correlated with 8-OHdG only 6 months after treatment (*p* = 0.005) and with 4-HNE in T1 and T2 (*p* = 0.017 and *p* = 0.001), whereas for the non-RA subjects, CPITN correlated with 8-OHdG (*p* = 0.023) and 4-HNE (0.043) at baseline and only with 8-OHdG 6 months after periodontal treatment (*p* = 0.001).

Other studies investigating serum concentrations of 8-OHdG in periodontitis have pointed out that they could be influenced by several systemic conditions—hyperlipidemia, rheumatoid arthritis, diabetes, and acute coronary syndrome [25,29,31,32]. Based on the aforementioned studies, we can conclude that local 8-OHdG levels are closely related to periodontitis, coexisting with some minor impact from systemic conditions, while systemic 8-OHdG levels are significantly influenced by general conditions and not so much by periodontal status.

In a review of the literature on the effects of multiple disease-modifying antirheumatic drugs (DMARDs) therapies on oxidative stress markers, the authors reveal an improvement in redox status following therapy with these drugs, either by decreasing oxidative stress markers or by increasing antioxidant capacity. This improvement in redox status may be correlated with the implementation of rheumatoid disease therapy [33].

Another recent systematic review that took into consideration the duration of anti-TNF therapy evaluated the effect on the periodontal status of rheumatoid arthritis subjects. Predominantly, the study revealed that periodontal health was improved after anti-TNF treatment. When considering the duration of therapy (6 weeks to 9 months), bleeding on probing (BOP) and gingival index (GI) were ameliorated after 6 weeks; however, probing pocket depth and clinical attachment level (CAL) improved after a longer period of time (3 months and 6 months, respectively). The authors concluded that anti-TNF therapy is favorable for both rheumatic joints but also for the periodontal tissues of arthritis-affected subjects [34].

Since our study included patients with a long history of biologic disease-modifying antirheumatic drug therapy that potentially decreases oxidative stress, we can conclude that laser and photoactivation adjuvant periodontal therapy brings undeniable additional benefits to oxidative status. Moreover, periodontal therapy managed to drop the analyzed oxidative stress markers in the RA group up to levels close to the non-RA group without any additional inflammatory and immunological load, especially for sites with adjuvant therapy; hence, the major benefit of these therapies in patients with an immune–inflammatory imbalance such as rheumatoid arthritis could be considered.

To our knowledge, this is the first clinical study that analyzes the effect of adjunctive periodontal treatment (diode laser and antimicrobial photodynamic therapy) in a split-mouth design on patients with periodontitis and rheumatoid arthritis. We recognized statistically significant outcomes (both in clinical periodontal parameters and in oxidative stress markers) in pockets treated with laser or antimicrobial photodynamic therapy when compared to sites treated only with scaling and root planning, thus proving the additional benefits of these therapies in the studied groups, especially in rheumatoid arthritis patients.

The oral cavity is continuously exposed to the action of endogenous and exogenous biological, chemical, and physical factors that are responsible for the excessive production of reactive oxygen species. ROS overproduction can disrupt the redox balance of the oral cavity, predisposing to oxidation and leading to oxidative damage of proteins, lipids, and genetic material. Recognition of exogenous sources of ROS and limiting exposure to ROS-generating factors may be one of the prophylactic measures to prevent oral and systemic diseases.

Our findings support the use of body fluids, and especially saliva and GCF, as tools for non-invasive diagnosis or monitoring of the evolution of periodontitis. The current data do not support the use of a single marker, but rather, a set to cover oxidative stress and also antioxidant capacity. Additionally, the low specificity of oxidative stress markers requires caution in interpreting the results, even if there are several markers used due to inter- and intra-individual variability [10].

Oxidative stress is a dynamic and complex phenomenon that occurs in rheumatoid arthritis and periodontitis and is involved in the pathogenesis of the disease in a complex way. Unfortunately, the real evidence is rather incongruent in what concerns the role of some molecules related to the regulatory process, and these discrepancies complicate the understanding of the involved mechanism. The variability and complexity of mechanisms for regulating oxidative stress in humans that are associated with genetic, epigenetic, age, gender, and dietary factors may explain these disparities. The results issued by our study suggest the use of several markers of oxidative stress as potential biomarkers for assessing disease activity and possibly prognosis.

## 5. Conclusions

At baseline, patients with RA had higher values of 8-OHdG compared to non-RA patients, and 4-HNE levels were similar in both studied groups. The analyzed oxidative stress markers decreased significantly following non-surgical periodontal therapy in both rheumatoid arthritis and systemically healthy patients. Out of the three periodontal disease treatment possibilities analyzed in our study, laser and photodisinfection yielded the best clinical and paraclinical outcomes.

## Figures and Tables

**Table 1:** Demographic characteristics of the RA group and the non-RA group at baseline for patients.

Parameters	All Cases (*n* = 52)	RA (*n* = 26)	Non-RA (*n* = 26)	Statistical Tests	*p*
Gender Female n (%) Male n (%)	32 (61.6%) 20 (38.4%)	19 (73.1%) 7 (26.9%)	13 (50.0%) 13 (50.0%)	Chi^2^ test	0.090
Age (y), mean ± SD	52.60 ± 12.65 (28−78)	51.02 ± 13.56 (28−78)	54.34 ± 11.50 (34−78)	F _ANOVA_ test	0.226
Area Urban Rural	29 (55.8%) 23 (44.2%)	15 (57.7%) 11 (42.3%)	14 (53.9%) 12 (46.1%)	Chi^2^ test	0.782
PD, mean ± SD	5.21 ± 0.87 (3.40−7.70)	5.40 ± 0.80 (3.40−7.30)	5.02 ± 0.91 (6.40−7.70)	F _ANOVA_ test	0.001
No. situs	3 × 52	3 × 26	3 × 26		
GI index 2 3 4	10 (6.4%) 84 (53.8%) 62 (39.7%)	5 (6.4%) 36 (46.2%) 37 (47.4%)	5 (6.4%) 48 (61.5%) 25 (32.1%)	Chi-square test Likelihood Ratio	0.132
PBI index 1 2 3 4	1 (0.6%) 30 (19.2%) 81 (51.9%) 44 (28.2%)	1 (1.3%) 24 (30.8%) 35 (44.9%) 18 (23.1%)	0 (0.0%) 6 (7.7%) 46 (59.0%) 26 (33.3%)	Chi-square test Likelihood Ratio	0.001
CPITN index 3 4	72 (46.2%) 84 (53.8%)	34 (43.6%) 44 (56.4%)	38 (48.7%) 40 (51.3%)	Chi-square test Likelihood Ratio	0.521
Mean number of teeth left	20 ± 3	18 ± 4	22 ± 1	F _ANOVA_ test	0.077

RA—rheumatoid arthritis + periodontal disease patients; non-RA—systemically healthy periodontal disease patients; PD—pocket probing depth; GI—gingival index; PBI—papillary bleeding index; CPITN—community periodontal index of treatment needs; *p* < 0.05 was considered statistically significant.

**Table 2:** Clinical periodontal indexes by treatment in RA and non-RA groups at baseline, T1, and T2, for individual sites.

Periodontal Clinical Indexes	RA (*n* = 78 Teeth)				non-RA (*n* = 78 Teeth)				RA vs. non-RA *p*
	C (*n* = 26)	L (*n* = 26)	PDT (*n* = 26)	*p*	C (*n* = 26)	L (*n* = 2 6)	PDT (n = 26)	*p*	
T0									
PD, mean±SD	5.87 ± 0.57	5.98 ± 0.79	6.02 ± 0.77	*0.726	5.38 ± 0.68	5.60 ± 0.94	5.70 ± 0.85	*0.378	*0.007 ^(C)^ *0.117 ^(L)^ *0.158 ^(P)^
GI index 2 3 4	2 (7.7%) 16 (61.5%) 8 (30.8%)	1 (3.8%) 10 (38.5%) 15 (57.7%)	2 (7.7%) 10 (38.5%) 14 (53.8%)	0.304	1 (3.8%) 19 (73.1%) 6 (23.1%)	2 (7.7%) 14 (53.8%) 10 (38.5%)	2 (7.7%) 15 (57.7%) 9 (34.6%)	0.666	0.643 ^(C)^ 0.345 ^(L)^ 0.350 ^(P)^
PBI index 1 2 3 4	0 (0.0%) 8 (30.8%) 14 (53.8%) 4 (15.4%)	1 (3.8%) 9 (34.6%) 9 (34.6%) 7 (26.9%)	0 (0.0%) 7 (26.9%) 12 (46.2%) 7 (26.9%)	0.592	0 (0.0%) 2 (7.7%) 19 (73.1%) 5 (19.2%)	0 (0.0%) 3 (11.5%) 13 (50.0%) 10 (38.5%)	(0.0%) 1 (3.8%) 14 (53.8%) 11 (42.3%)	0.291	0.094 ^(C)^ 0.122 ^(L)^ 0.047 ^(P)^
CPITN index 3 4	12 (46.2%) 14 (53.8%)	12 (46.2%) 14 (53.8%)	10 (38.5%) 16 (61.5%)	0.811	18 (69.2%) 8 (30.8%)	11 (42.3%) 15 (57.7%)	9 (34.6%) 17 (65.4%)	0.030	0.080 ^(C)^ 0.500 ^(L)^ 0.500 ^(P)^
**T1**									
PD, mean±SD	5.29 ± 0.61	5.21 ± 0.75	5.23 ± 0.70	*0.913	4.82 ± 0.70	4.96 ± 0.96	5.06 ± 0.73	*0.563	*0.013 ^(C)^ *0.293 ^(L)^ *0.396 ^(P)^
T1 vs. T0	*0.001	*0.001	*0.001	-	*0.012	*0.050	*0.014	-	
GI index 1 2 3 4	1 (3.8%) 12 (46.2%) 12 (46.2%) 1 (3.8%)	6 (23.1%) 14 (53.8%) 5 (19.2%) 1 (3.8%)	5 (19.2%) 12 (46.2%) 9 (34.6%) 0 (0.0%)	0.166	2 (7.7%) 18 (69.2%) 6 (23.1%) 0 (0.0%)	3 (11.5%) 15 (57.7%) 8 (30.8%) 0 (0.0%)	4 (15.4%) 14 (53.8%) 8 (30.8%) 0 (0.0%)	0.809	0.174 ^(C)^ 0.371 ^(L)^ 0.850 ^(P)^
T1 vs. T0	0.001	0.001	0.001	-	0.001	0.001	0.001	-	
PBI index 0 1 2 3	0 (0.0%) 4 (15.4%) 14 (53.8%) 8 (30.8%)	2 (7.7%) 8 (30.8%) 15 (57.7%) 1 (3.8%)	1 (3.8%) 8 (30.8%) 16 (61.5%) 1 (3.8%)	0.031	0 (0.0%) 0 (0.0%) 24 (92.3%) 2 (7.7%)	0 (0.0%) 7 (26.9%) 15 (57.7%) 4 (15.4%)	0 (0.0%) 4 (15.4%) 17 (65.4%) 5 (19.2%)	0.009	0.002 ^(C)^ 0.190 ^(L)^ 0.128 ^(P)^
T1 vs. T0	0.001	0.001	0.001	-	0.001	0.001	0.001	-	
CPITN index 1 2 3 4	0 (0.0%) 2 (7.7%) 22 (84.6%) 2 (7.7%)	0 (0.0%) 5 (19.2%) 16 (61.5%) 5 (19.2%)	0 (0.0%) 5 (19.2%) 18 (69.2%) 3 (11.5%)	0.398	0 (0.0%) 12 (46.2%) 13 (50.0%) 1 (3.8%)	1 (3.8%) 19 (73.1%) 6 (23.1%) 0 (0.0%)	1 (3.8%) 12 (46.2%) 13 (50.0%) 0 (0.0%)	0.153	0.005 ^(C)^ 0.001 ^(L)^ 0.025 ^(P)^
T1 vs. T0	0.001	0.001	0.001	-	0.001	0.001	0.001	-	
**T2**									
PD, mean±SD	5.03 ± 0.61	4.96 ± 0.73	4.99 ± 0.71	*0.936	4.51 ± 0.68	4.56 ± 0.94	4.62 ± 0.80	*0.877	*0.005 ^(C)^ *0.093 ^(L)^ *0.085 ^(P)^
T2 vs. T0	*0.001	*0.001	*0.001	-	*0.001	*0.001	*0.001	-	
GI index 1 2 3	5 (19.2%) 11 (42.3%) 10 (38.5%)	12 (46.2%) 12 (46.2%) 2 (7.7%)	12 (46.2%) 14 (53.8%) 0 (0.0%)	0.001	1 (3.8%) 18 (69.2%) 7 (26.9%)	9 (34.6%) 16 (61.5%) 1 (3.8%)	11 (42.3%) 13 (50.0%) 2 (7.7%)	0.002	0.076 ^(C)^ 0.511 ^(L)^ 0.240 ^(P)^
T2 vs. T0	0.001	0.001	0.001	-	0.001	0.001	0.001	-	
PBI index 0 1 2 3	0 (0.0%) 3 (11.5%) 16 (61.5%) 7 (26.9%)	4 (15.4%) 11 (42.3%) 11 (42.3%) 0 (0.0%)	6 (23.1%) 11 (42.3%) 8 (30.8%) 1 (3.8%)	0.001	0 (0.0%) 10 (38.5%) 11 (42.3%) 5 (19.2%)	3 (11.5%) 7 (26.9%) 14 (53.8%) 2 (7.7%)	2 (7.7%) 9 (34.6%) 15 (57.7%) 0 (0.0%)	0.052	0.073 ^(C)^ 0.243 ^(L)^ 0.119 ^(P)^
T2 vs. T0	0.007	0.007	0.007	-	0.001	0.001	0.001	-	
CPITN index 0 1 2 3	0 (0.0%) 0 (0.0%) 7 (26.9%) 19 (73.1%)	1 (3.8%) 2 (7.7%) 9 (34.6%) 14 (53.8%)	0 (0.0%) 2 (7.7%) 11 (42.3%) 13 (50.0%)	0.265	0 (0.0%) 0 (0.0%) 17 (65.4%) 9 (34.6%)	0 (0.0%) 5 (19.2%) 18 (69.2%) 3 (11.5%)	0 (0.0%) 7 (26.9%) 17 (65.4%) 2 (7.7%)	0.003	0.012 ^(C)^ 0.004 ^(L)^ 0.001 ^(P)^
T2 vs. T0	0.001	0.001	0.001	-	0.001	0.001	0.001	-	

T0—before periodontal treatment; T1—after 3 therapy sessions; T2—6 months after the end of periodontal therapy. C—control: scaling and root planning (SRP) sites; L—SRP + laser sites; PDT—SRP + photodisinfection sites. RA—rheumatoid arthritis + periodontal disease patients; non-RA—systemically healthy periodontal disease patients; PD—pocket probing depth; GI—gingival index; PBI—papillary bleeding index; CPITN—community periodontal index of treatment needs; p Chi-square test Likelihood Ratio or * *p* FANOVA test. *p* < 0.05 was considered statistically significant.

**Table 3:** Mean values of 8-OHdG.

Time of Sampling	RA Group (*n* = 78 Teeth)			*p* *	non-RA Group (*n* = 78 Teeth)			*p* *	*p* * _RA vs. non-RA_		
	C(*n* = 26)	L(*n* = 26)	PDT (*n* = 26)		C (*n* = 26)	L (*n* = 26)	PDT(*n* = 26)		C	L	PDT
**T0**	40.60 ± 8.13	41.35 ± 9.01	41.65 ± 9.30	0,908	32.70 ± 5.07	31.41 ± 6.01	31.55 ± 4.77	0.663	0.001	0.001	0.001
**T1**	15.18 ± 2.68	5.97 ± 1.28	6.19 ± 1.43	0.001	14.96 ± 2.44	6.18 ± 0.85	6.30 ± 0.97	0.001	0.763	0.479	0.752
P _T1 vs. T0_	0.001	0.001	0.001	-	0.001	0.001	0.001	-	-	-	-
**T2**	8.04 ± 1.08	3.46 ± 0.53	3.46 ± 0.56	0.001	8.29 ± 1,29	3.49 ± 0.17	3.42 ± 0.32	0.001	0.445	0.779	0.765
P _T2 vs. T0_ P _T2 vs. T1_	0.001 0.001	0.001 0.001	0.001 0.001	-	0.001 0.001	0.001 0.001	0.001 0.001	-	-	-	-

T0—before periodontal treatment; T1—after 3 therapy sessions; T2—6 months after the end of periodontal therapy. C—control: scaling and root planning (SRP) sites; L—SRP + laser sites; PDT—SRP + photodisinfection sites; RA—rheumatoid arthritis + periodontal disease patients; non-RA—systemically healthy periodontal disease patients; * *p* FANOVA test. *p* < 0.05 was considered statistically significant.

**Table 4:** Mean values of 4-HNE.

Time of Sampling	RA Group (*n* = 78 Teeth)			*p* *	non-RA Group (*n* = 78 Teeth)			*p* *	*p* * _RA vs. non-RA_		
	C (*n* = 26)	L (*n* = 26)	PDT (*n* = 26)		C(*n* = 26)	L (*n* = 26)	PDT (*n* = 26)		C	L	PDT
**T0**	33.32 ± 4.05	33.20 ± 4.22	33.58 ± 3.25	0.934	32.52 ± 1.57	32.27 ± 3.16	32.42 ± 2.85	0.940	0.357	0.372	0.177
**T1**	14.35 ± 3.19	6.48 ± 3.18	7.29 ± 3.86	0.001	28.53 ± 1.28	24.89 ± 1.36	24.41 ± 2.17	0.001	0.001	0.001	0.001
P _T1 vs. T0_	0.001	0.001	0.001	-	0.001	0.001	0.001	-	-	-	-
**T2**	3.18 ± 0.48	1.81 ± 0.66	1.48 ± 0.57	0.001	18.49 ± 1.15	13.05 ± 2.92	12.25 ± 2.08	0.001	0.001	0.001	0.001
P _T2 vs. T0_ P _T2 vs. T1_	0.001 0.001	0.001 0.001	0.001 0.001	-	0.001 0.001	0.001 0.001	0.001 0.001	-	-	-	-

T0—before periodontal treatment; T1—after 3 therapy sessions; T2—6 months after the end of periodontal therapy. C—control: scaling and root planning (SRP) sites; L—SRP + laser sites; PDT—SRP + photodisinfection sites; RA—rheumatoid arthritis + periodontal disease patients; non-RA—systemically healthy periodontal disease patients; * *p* FANOVA test. *p* < 0.05 was considered statistically significant.

**Table 5:** Association between 8-OhdG and 4-HNE and GI, PBI, or CPITN.

Index	RA Group				non-RA Group			
	T0	T1	T2	*p* for F _ANOVA_ Test	T0	T1	T2	*p* for F _ANOVA_ Test
8-OHdG, mean±SD								
GI 1 2 3 4	- 39.54 ± 3.06 41.63 ± 8.66 40.01 ± 8.72	7.14 ± 2.82 8.87 ± 4.79 10.29 ± 4.95 10.30 ± 8.34	4.08 ± 1.73 4.93 ± 2.19 7.35 ± 2.35 -	0.001 0.001 0.001 0.001	- 30.74 ± 3.96 31.37 ± 4.89 33.11 ± 6.12	8.46 ± 4.52 9.71 ± 4.72 8.23 ± 3.68 -	3.62 ± 0.71 5.36 ± 2.55 6.76 ± 2.65 -	0.001 0.001 0.001 -
p F _ANOVA_ test	0.870	0.268	0.001	-	0.366	0.385	0.001	-
4-HNE, mean ± SD								
GI 1 2 3 4	- 32.96 ± 1.59 32.86 ± 4.08 33.91 ± 3.76	9.94 ± 4.79 9.06 ± 4.96 9.44 ± 5.11 11.15 ± 4.74	1.84 ± 0.74 2.12 ± 0.94 3.03 ± 0.86 -	0.001 0.001 0.001 0.001	- 31.76 ± 3.16 32.19 ± 2.33 32.95 ± 2.94	24.86 ± 3.15 26.25 ± 2.89 25.74 ± 2.51 -	14.07 ± 3.04 15.45 ± 3.17 16.93 ± 2.85 -	0.001 0.007 0.007 -
p F _ANOVA_ test	0.491	0.904	0.001	-	0.423	0.272	0.050	
8-OHdG, mean ± SD								
PBI 0 1 2 3 4	- 34.90 ± 0.0 40.90 ± 7.16 41.63 ± 9.75 41.09 ± 9.06	6.40 ± 0.82 8.11 ± 3.61 8.82 ± 4.95 13.26 ± 4.26 -	3.58 ± 0.36 3.90 ± 1.58 5.55 ± 2.43 7.68 ± 1.99 -	0.001 0.001 0.001 0.001 -	- - 28.55 ± 4.44 31.23 ± 4.67 33.81 ± 5.94	- 5.99 ± 0.77 10.08 ± 4.75 7.55 ± 2.77 -	3.30 ± 0.21 5.25 ± 2.30 4.69 ± 2.13 7.83 ± 3.27 -	- 0.995 0.001 0.001 -
p F _ANOVA_ test	0.892	0.017	0.001	-	0.035	0.007	0.003	
4-HNE, mean ± SD								
PBI 0 1 2 3 4	- 32.90 ± 0.0 32.97 ± 4.21 33.27 ± 3.61 34.11 ± 3.89	7.37 ± 3.18 9.37 ± 5.14 8.99 ± 4.90 11.73 ± 4.71 -	1.60 ± 0.52 1.82 ± 0.77 2.28 ± 0.96 3.38 ± 0.42 -	0.001 0.001 0.001 0.001 -	- - 31.35 ± 1.92 32.23 ± 2.21 32.95 ± 3.22	- 24.63 ± 2.06 26.32 ± 2.42 25.33 ± 2.70 -	14.22 ± 1.99 15.78 ± 2.36 15.03 ± 2.67 15.47 ± 7.42 -	- 0.049 0.001 0.001 -
p F _ANOVA_ test	0.815	0.534	0.038	-	0.309	0.075	0.692	
8-OHdG, mean±SD								
CPITN 0 1 2 3 4	- - - 39.16 ± 8.75 42.77 ± 8.46	- - 7.13 ± 4.19 9.76 ± 4.88 7.85 ± 3.62	3.10 ± 0.0 3.40 ± 0.36 4.44 ± 2.05 5.49 ± 2.43 -	- - 0.001 0.001 0.001	- - - 30.53 ± 5.27 33.18 ± 5.01	- 5.30 ± 0.14 8.85 ± 4.47 9.85 ± 4.46 7.40 ± 0.0	- 14.91 ± 1.69 15.15 ± 3.09 15.99 ± 4.39 -	- 0.001 0.041 0.001 0.001
p F _ANOVA_ test	0.069	0.142	0.050	-	0.023	0.454	0.001	
4-HNE, mean±SD								
CPITN 0 1 2 3 4	- - - 32.21 ± 5.10 34.26 ± 2.06	- - 7.99 ± 4.71 9.73 ± 5.18 9.08 ± 3.38	2.20 ± 0.0 1.43 ± 0.56 1.86 ± 0.82 2.40 ± 0.97 -	- - 0.001 0.001 0.001	- - - 31.80 ± 2.41 32.98 ± 2.64	- 22.90 ± 1.12 25.78 ± 2.36 26.23 ± 2.52 29.60 ± 0.0	- 3.53 ± 0.31 4.93 ± 2.24 6.92 ± 2.96 -	- 0.001 0.001 0.001 0.112
p F _ANOVA_ test	0.017	0.534	0.001	-	0.043	0.116	0.629	

T0—before periodontal treatment; T1—after 3 therapy sessions; T2—6 months after the end of periodontal therapy. C—control: scaling and root planning (SRP) sites; L—SRP + laser sites; PDT—SRP + photodisinfection sites; RA—rheumatoid arthritis + periodontal disease patients; non-RA—systemically healthy periodontal disease patients; PD—pocket probing depth; GI—gingival index; PBI—papillary bleeding index; CPITN—community periodontal index of treatment needs; 8-OHdG—8-hydroxy-2’-deoxyguanosine; 4-HNE—hydroxynonenal. *p* < 0.05 was considered statistically significant.

## Data Availability

The data used to support the findings of this study are available from the corresponding author upon reasonable request.

## Supplementary Materials

The following are available online at https://www.mdpi.com/2076-3921/10/2/226/s1; Figure S1: Correlations between 8OHdG and PD in RA and nonRA groups; Figure S2: Correlations between 4HNE and PD in RA and nonRA groups.
