# Supplementary material for: Evaluation of Oxidative Stress before and after Using Laser and Photoactivation Therapy as Adjuvant of Non-Surgical Periodontal Treatment in Patients with Rheumatoid Arthritis
Source: Antioxidants (Basel). 2021 Feb 3;10(2):226. doi: 10.3390/antiox10020226 (PMC7913189; doi:10.3390/antiox10020226)
Supplement: Supplementary file 1 [file antioxidants-10-00226-s001.pdf]

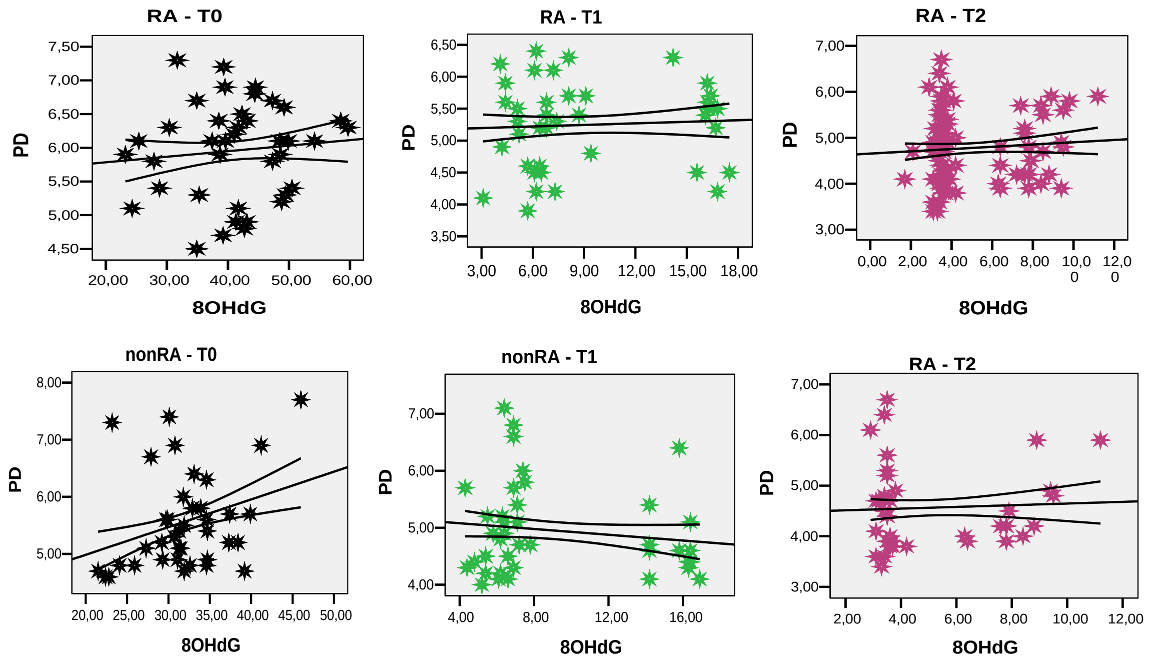

Figure 1. Correlations between 8OHdG and PD in RA and nonRA groups.

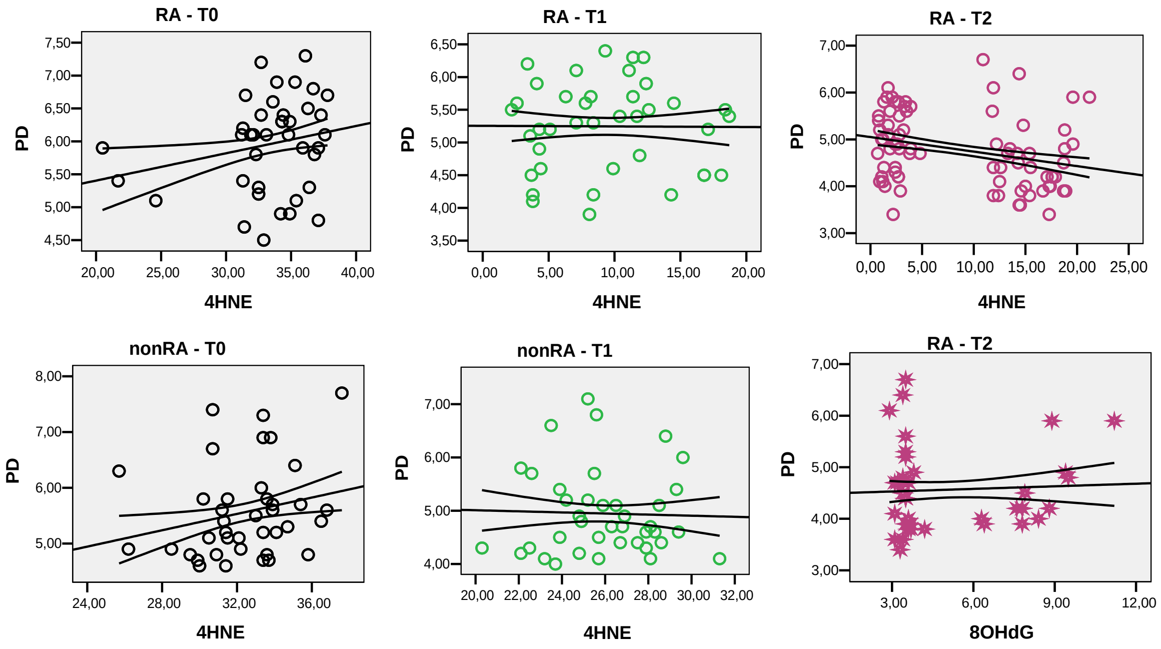

Figure 2. Correlations between 4HNE and PD in RA and nonRA groups.
